# Topics of the Month

**Published:** 1893-07

**Authors:** 


### TOPICS OF THE MONTH.

NATIONAL QUARANTINE.—REPORT OF A COMMITTEE OF THE NEW YORK ACADEMY OF MEDICINE.—Dr. D. B. St. John Roosa, who presided at the meeting of the Academy of Medicine, held June 20, 1893, was agreeably surprised at the large attendance, despite the great heat. It was a special meeting called "at the request of ten members of the National Quarantine Committee, to receive a report from the same committee." This is the report, which was read by Dr. Derby:

While we believe that the most certain protection of this or any other civilized country against the ravages of any infectious disease lies in the practice of carefully planned internal sanitation, we yet are convinced that, for the present at least, an intelligently rigorous system of maritime quarantine is necessary in this country. So far as the protection of the country against Asiatic cholera during the coming Summer is concerned, the legislative bodies in whose hands the responsibility lays have decided, for better or for worse, upon their plan; and upon the authorities on whom the delegated power, such as it is, was vested, now rests the task of execution.

Our investigation of this subject has convinced us that the interest of the public health of the country demands the establishment, at an early day, of a National Bureau of Health, under whose supervision and control, together with other matters concerning the public health, them aritime quarantine system should be placed. The duties of such a board, in addition to those involved in a maritime quarantine, should be to sustain and encourage research in matters concerning the causes and prevention of disease, to obtain and disseminate information on matters affecting public health, to formulate and enforce such rules and regulations as may be necessary to prevent the introduction of contagious or infectious diseases and the spread of the same within the territory of the United States, to advise the several departments of the government, the executives of the several States, and the commissioners of the District of Columbia, on all questions submitted to them relating to the public health, and to tender such advice whenever in the opinion of the board such counsel may be conducive to the public weal.

After deliberation, the National Quarantine Commission of the Academy of Medicine has agreed that in order to secure the objects for which it was appointed, it seems necessary that a National Bureau of Health should be organized—quarantine being a function of the bureau—and it desires to be authorized by the academy to take such steps as it may consider necessary to carry out this project. Respectfully submitted,

I. G. THOMAS, *Chairman.*
RICHARD H. DERBY, *Secretary.*

Dr. T. Gaillard Thomas spoke in support of the suggestions contained in the report.

Dr. W. W. McGregor, of Laredo, Texas, said that in his opinion the matter of quarantine should be under a thoroughly systematised National Quarantine Board.

Dr. A. D. Chapin moved, and Dr. J. D. Bryant seconded a motion calling for the adoption of the report and the carrying out of the suggestions therein contained.

The Chairman said that they all had confidence in the committee, and that it could be intrusted with any work that the academy in its wisdom saw fit to empower it to carry out.

Dr. Jacobi wanted to know whether they would expect the committee to report before anything very important was really done.

Dr. Roosa said:

It will not be necessary for the committee to report until the members themselves deem it proper to do so. It would be belittling them to say that they would do nothing until we meet again in October. As a matter of courtesy to this body, I have no doubt that they will give information, from time to time, of what they are doing, and if the Academy sees fit to correct or reverse their action, it has the right and the power to do so.

The resolution was immediately agreed to.—*New York Tribune.*

---

THE Health Department having failed to suppress the church-bell nuisance, it would seem to be in order to appeal to the Department of Police to take action in the premises. The church bells at 5.00 and 6.00 A. M. are a far greater nuisance, and more disturbing to the sick, than the hurdy-gurdies after 8.00 P. M.

The New York Physicians' Mutual Aid Association has established its headquarters permanently in the capacious New York Academy of Medicine building, 17, 19, and 21 W. Forty-third street, New York City. We have on several occasions referred to the good work this association is doing, and we desire to further emphasize the subject at this time. This association furnishes a certificate payable at death for $1,000, the cost of which is but a trifle. There is no other way that insurance can be afforded at so little cost and with such absolute safety. The membership is steadily increasing, but it should be advanced to 2,000 in number, in which case the assessments would be reduced one-half. Every physician residing in the State of New York should interest himself to secure a member. The address of the secretary is Dr. J. E. H. Nichols, 4 E. Forty-third street, New York, N. Y.

---

The first annual report of the Sheppard Asylum and Hospital for Mental Diseases, located near Baltimore, Md., is a model of its kind. This asylum was founded through the generosity of the late Mr. Moses Sheppard, of Baltimore, and is intended to afford the most advanced accommodation and treatment for those who are mentally afflicted. The institution is under the medical superintendence of Dr. Edward N. Brush, who is also physician-in-chief, and who will be remembered by his many friends in Buffalo, as a former practitioner of medicine in this city, and at one time editor of the Journal.

The report is beautifully printed, and handsomely illustrated by photographic reproductions of the buildings, grounds, and interior views. The Journal begs to tender to Dr. Brush its congratulations upon his success in his present field of labor, and to congratulate the trustees of the Sheppard Asylum upon securing his valuable services as chief medical officer of that institution.

---

We beg to call the attention of our readers to an interesting paper by Prof. Isaac N. Love, of St. Louis, which occupies the first place in this number of the Journal. The subject upon which he discourses, namely, Children's Rights, is one of great importance, and we are not aware that it has ever been more ably handled than by the distinguished author who makes his bow to our readers as a contributor on this occasion. The address is in the nature of a valedictory to the graduating class, and his closing words, addressed more especially to the graduates themselves, also deserves a careful reading. His remarks relating to the code are especially significant, in view of the fact that Dr. Love has just been elected Vice-President of the American Medical Association, and in some sense has foreshadowed the change of sentiment which is rapidly taking place in that body on the much controverted code of ethics.

---

Dr. C. N. Hoagland, of Brooklyn, a physician already widely known for his wise beneficence in the promotion of medical science, has made a generous offer to the medical profession of the city of his residence. As our readers are well aware, the Hoagland Laboratory is a monument to his interest both in medicine and in the city of his home. But Dr. Hoagland does not stop at one benefaction, for now he proposes to give $50,000 for the erection of a building which shall serve as a headquarters for all the medical societies in Brooklyn, provided that an equal sum for the same purpose is raised by the medical profession of that city. No doubt this generous offer will be accepted by the several societies in interest. We understand that already committees have been appointed to consider this magnanimous offer. It is a pity that some public-spirited citizen of Buffalo, of whom we are proud to say there are many, could not be made to see the propriety of a like contribution for the promotion of medical science, and to the everlasting credit of the donor and the city. We wish the example of Dr. Hoagland might stimulate some such similar action in Buffalo.

---

The Smithsonian Institution, at Washington, has issued a circular concerning the Hodgkins fund prizes. These are four in number, and were established by Thomas George Hodgkins, Esq., of Setaukett, N. Y., to increase and diffuse more interest and knowledge in regard to the nature and properties of atmospheric air in connection with the welfare of mankind. The Smithsonian Institution announces the following prizes to be awarded on or after July 1, 1894, should satisfactory papers be offered in competition:

1. A prize of $10,000 for a treatise embodying some new and important discovery in regard to the nature or properties of atmospheric air. These properties may be considered in their bearing upon any or all of the sciences—*e. g.*, not only in regard to meteorology, but in connection with hygiene, or with any department whatever of biological or physical knowledge.

2. A prize of $2,000 for the most satisfactory essay upon—

(*a*) The known properties of atmospheric air considered in their relationships to research in every department of natural science, and the importance of a study of the atmosphere considered in view of these relationships.

(*b*) The proper direction of future research in connection with the imperfections of our knowledge of atmospheric air, and of the connection of that knowledge with our sciences.

The essay, as a whole, should tend to indicate the path best calculated to lead to worthy results in connection with the future administration of the Hodgkins foundation.

3. A prize of $1,000 for the best popular treatise upon the atmospheric air, its properties and relationships (including those to hygiene, physical and mental). This essay need not exceed 20,000 words in length; it should be written in simple language, and be suitable for publication for popular instruction.

4. A medal will be established, under the name of the Hodgkins Medal of the Smithsonian Institution, which will be awarded annually or biennially, for important contributions to our knowledge of the nature and properties of atmospheric air, or for practical applications of our existing knowledge of them to the welfare of mankind. This medal will be gold, and will be accompanied by a duplicate impression in silver or bronze.

All communications in regard to the Hodgkins fund prizes should be addressed to S. P. Langley, Esq., Secretary of the Smithsonian Institution, Washington, D. C.

---

THE fourth bulletin of the Harvard Medical School Association was issued in May, 1893. Like the bulletins that have preceded it, it is a model of its kind. It contains a list of the officers of the Association, a report of the meeting of the Council, and a number of valuable papers. It is intended, as stated in the prefatory note, to give to the graduates of the school a brief account of certain new methods of teaching which have arisen in the various departments of Harvard Medical School. It is beautifully printed on heavy paper, and contains two handsome photographic illustrations. Applications for this valuable bulletin should be made to the Secretary, Dr. Robert Williamson Lovett, 379 Boylston street, Boston, Mass.

